# Multimorbidity patterns and their associations with demographic characteristics, behavioral factors, and urban–rural residence: A latent class analysis in the Thai population

**DOI:** 10.1371/journal.pone.0354347

**Published:** 2026-07-29

**Authors:** Pavitra Jindahra, Stefano Starita, Pinnaree Tea-makorn

**Affiliations:** Artificial Intelligence and Data Analytics Center, Sasin School of Management, Chulalongkorn University, Pathumwan, Bangkok, Thailand; University of Naples Federico II: Universita degli Studi di Napoli Federico II, ITALY

## Abstract

Multimorbidity, defined as the presence of two or more chronic conditions, is a growing public health concern. Understanding disease clustering and risk profiles is crucial for prevention and planning purposes. This study analyzed data from the National Health Survey in Thailand, which included 142,753 participants. Latent class analysis investigated nine non-communicable diseases, while multinomial logistic regression explored the characteristics associated with class membership. Four distinct classes were identified: low burden, hypertension–metabolic, predominant hypertension, and high multimorbidity. Advancing age revealed an increased probability of membership in all multimorbidity classes. Females exhibited a higher propensity to belong to the hypertension–metabolic and predominant hypertension classes. Urban residency was associated with an elevated probability of membership in the hypertension–metabolic and high-multimorbidity classes. The body mass index displayed significant variations across classes. Overweight and obesity were linked to the hypertension–metabolic and predominant hypertension classes, with obesity also associated with the high multimorbidity class. Underweight individuals demonstrated a reduced likelihood of membership in the hypertension–metabolic class but an increased likelihood of membership in the predominant hypertension class. Smoking was correlated with a higher probability of membership across classes, particularly among current smokers. Dietary behaviors were distinctly associated across classes. Frequent consumption of high-fat and instant foods was linked to the hypertension–metabolic class, whereas instant food consumption was also associated with the predominant hypertension class. Fruit consumption was correlated with a higher probability of membership in the hypertension–metabolic class, whereas vegetable consumption was associated with a reduced likelihood of membership in the predominant hypertension class. Moderate consumption of non-alcoholic sugary drinks was associated with a lower probability of hypertension–metabolic class membership. These findings underscore the heterogeneity of multimorbidity patterns in Thailand and delineate the demographic, behavioral, and residential profiles associated with non-communicable disease clustering.

## Introduction

Multimorbidity, defined as the coexistence of two or more chronic diseases, is a major health concern worldwide. Its prevalence ranges from 3.5% to 98.5%, depending on population characteristics and definitions [1–3]. In Thailand, international and regional comparisons suggest that the prevalence of multimorbidity among older adults is lower than that reported in many high-income regions [4–7]. The burden of multimorbidity strains healthcare systems by complicating treatment, increasing costs, and challenging single-disease care models. Therefore, integrated approaches that address multiple conditions are essential, especially in developing countries where healthcare infrastructure is less robust.

Research shows that multimorbidity patterns vary across regions and are shaped by sociocultural, environmental, and health system contexts. In Europe and North America, patterns cluster around cardiovascular, metabolic, and mental health conditions, with variations influenced by lifestyle and health care delivery [8–12]. In Spain, three multimorbidity patterns have been linked to sociodemographic factors [12], whereas in Denmark, complex multimorbidity is more common among older women and is associated with behavioral and healthcare differences [10]. In Asia, common clusters include cardiovascular, metabolic, and mental health patterns [13–14]. In China, multimorbidity clusters include vascular, respiratory, and multisystem conditions [15], whereas Taiwanese studies have identified cardiometabolic, respiratory, and gastrointestinal linkages [16]. A systematic review of low- and middle-income countries suggested the frequent coexistence of cardiometabolic and mental health disorders, reflecting global overlaps and regional distinctions [17]. Studies from Africa have pointed to unique combinations, such as cardiorespiratory clusters, emphasizing local health conditions [18].

These findings underscore that while cardiovascular and metabolic diseases dominate globally, specific combinations vary across different settings. Understanding these patterns is vital for tailoring healthcare strategies and recognizing modifiable risk factors that contribute to disease onset and progression, such as physical inactivity, poor diet, and smoking. Moreover, awareness of the economic and quality of life burdens associated with multimorbidity patterns supports better resource allocation and policy planning [12].

In Thailand, demographic shifts and lifestyle changes have accelerated the prevalence of multimorbidity in recent years. Longer life expectancy and population aging have increased the burden of chronic conditions, whereas sedentary behavior and dietary transitions have further increased the prevalence of non-communicable diseases (NCDs) [17]. This convergence highlights the urgent need for targeted interventions that address the behavioral and environmental drivers of multimorbidity in Thailand.

Moreover, a range of non-communicable diseases is frequently observed in the adult population. According to data from the 2021 Thailand Health Behavior of Population Survey conducted by the National Statistical Office, hypertension is the most prevalent condition, affecting approximately 16.3% of individuals aged ≥ 15 years. This was followed by diabetes or elevated blood glucose levels (8.1%) and hyperlipidemia or elevated cholesterol levels (7.5%). Additional chronic conditions included osteoarthritis (3.8%), cardiovascular disease (1.7%), stroke (1.1%), and chronic respiratory diseases, such as chronic obstructive pulmonary disease and asthma (1.0%). Cancer accounts for approximately 0.6% of cases, whereas depression is reported by approximately 0.4% of the population. The concurrent presence of these chronic conditions in individuals highlights the necessity of examining their clustering as multimorbidity patterns within the Thai population.

While the recognition of multimorbidity has increased across countries, critical knowledge gaps persist in Southeast Asia. Most studies have been limited to prevalence estimates in clinical populations [19–20], with little exploration of how sociocultural and economic contexts shape multimorbidity. Research has focused on older adults, neglecting younger populations, even though multimorbidity affects individuals throughout their lives [21]. Moreover, behavioral and environmental determinants remain understudied as potential, modifiable risk factors [22]. Evidence on diet shows mixed associations with multimorbidity, highlighting the need for further research [23]. This lack of context-specific evidence limits our understanding and hinders the development of effective population-tailored public health strategies [5,24].

This study examined multimorbidity patterns in Thailand using a diverse sample aged 15–106 years. It investigated associations with key demographic, biological, behavioral, and residential factors, including age, gender, body mass index (BMI), smoking, dietary habits, and urban-rural residence. This study elucidated distinct patterns of multimorbidity and their associated characteristics, thereby identifying population subgroups with an elevated disease burden and the traits linked to these higher-risk clusters.

Insights from this study illuminate multimorbidity patterns in Southeast Asia, where research remains scarce, and enrich the global discourse by demonstrating how modifiable risk factors interact with sociocultural and economic conditions. This study expands the evidence base and deepens the understanding of multimorbidity in ways that can inform regional and global health strategies

## Materials and methods

### Methods

This study conducted a cross-sectional secondary analysis of data derived from the 2021 Thailand Health Behavior of Population Survey, administered by the National Statistical Office, Ministry of Digital Economy and Society, Thailand. Latent class analysis (LCA) was employed to delineate the patterns of NCDs and their associated demographic and behavioral characteristics within the Thai population. The survey gathered data on NCDs and key variables, including demographic characteristics (age and gender), biological factors (BMI), residential location (living area: municipal vs. non-municipal), and behavioral factors (dietary and smoking behaviors) from individuals aged 15–106 years.

The dataset is publicly accessible, fully anonymized, and devoid of personally identifiable information. Ethical approval and informed consent were obtained from the original survey administrators, and this secondary analysis was exempt from the institutional ethical review. Data were accessed in February 2024 for research. The authors did not have access to any information that could identify individual participants during or after data analysis.

### Study population

This survey included households and individuals residing in municipal and non-municipal areas across 77 provinces in Thailand, excluding foreign nationals in embassies or international organizations with diplomatic privileges. Municipal areas established by the Thai Royal Decree of the 1953 Municipality Act encompass cities, towns, and sub-districts with better access to healthcare, education, and infrastructure. Municipal cities must have 50,000 people and a density of 3,000 per square kilometer while generating sufficient tax revenues. Towns require a minimum population of 10,000 and a density of 3,000 per square kilometer, with adequate tax revenue for operations. Sub-district municipal areas need at least 7,000 residents and a density of 1,500 people per square kilometer. Areas that did not meet these criteria were classified as non-municipal. The study sample included 150,000 individuals from 84,000 households across the country. After excluding those with missing data, 142,753 participants were included in the study.

### Sampling design

The survey used a stratified two-stage sampling design. Provinces were designated as strata, with municipal and non-municipal areas as substrata. Enumeration areas served as primary sampling units, and the households served as secondary sampling units. A sampling frame was created by listing the households and building structures in each area. Households were organized by size based on the number of members in each household. The household sample was determined through systematic random sampling by selecting 16 households from municipal and non-municipal areas. Data were collected through personal interviews conducted between February 1 and May 31, 2021.

### Measures

Research has identified demographic, socioeconomic, and behavioral factors as contributors to the development of multimorbidity [23]. This study focuses on NCDs and their associated risk factors, including demographic characteristics such as age and gender, biological factors such as body mass index (BMI), residential location, and behavioral factors such as dietary and smoking behaviors. These variables were selected based on their established associations with NCDs in the epidemiological literature [23] and their relevance to the study’s objective of examining the demographic, behavioral, and residential factors associated with multimorbidity patterns.

NCDs: This study investigated nine NCDs: hypertension, diabetes mellitus, hyperlipidemia (high cholesterol), cardiovascular disease, stroke, chronic respiratory disease, cancer, depression, and osteoarthritis. The presence of NCDs was ascertained through self-reported physician diagnoses. Participants were asked whether they had ever been diagnosed with any of the aforementioned conditions by a physician.

Age: Numerous studies have shown a strong association between age, multimorbidity burden, and NCDs [25]. As the population ages, the probability of developing multiple chronic conditions increases [26–27], highlighting the necessity of comprehending age-related patterns of multimorbidity.

Gender: Gender differences are important because of their impact on multimorbidity patterns and health outcomes. Females frequently experience distinct health risks and outcomes compared with males [28]. Numerous studies have demonstrated that women generally exhibit a higher prevalence of multimorbidity than men do [15,29–31]. However, evidence regarding the relationship between gender and multimorbidity remains inconclusive. While some studies have reported no difference in the prevalence of multimorbidity between men and women [32], others have indicated a higher prevalence among men [22].

BMI: The Body Mass Index (BMI), a biological metric, was determined by dividing an individual’s weight in kilograms by the square of their height in meters (BMI = kg/m²). Respondents self-reported their height and weight based on their most recent measurements in the past 30 days. BMI classifications were as follows: underweight (BMI < 18.5), normal weight (18.5 ≤ BMI < 23), overweight (23 ≤ BMI < 25), and obese (BMI ≥ 25). The global epidemic of non-communicable diseases (NCDs) is closely linked to the rising prevalence of overweight and obesity, particularly in developing nations. The global obesity epidemic has substantially contributed to the increasing burden of NCDs, especially in developing countries [33–34].

Living area: Urbanization facilitates improved access to healthcare and the promotion of health resources; however, it also tends to increase the prevalence of unhealthy behaviors, such as sedentary lifestyles and the consumption of processed foods [35–36], which elevate the risk of multimorbidity. In contrast, rural areas may preserve healthier traditional diets but often experience limited dietary diversity and insufficient healthcare infrastructure, contributing to distinct patterns of clustered NCDs. Living areas were classified as urban for individuals residing in municipal areas and rural for those residing in non-municipal areas.

Dietary behaviors: Numerous studies have underscored the strong association between dietary habits and the risk of developing NCDs [37]. Diets characterized by high consumption of processed foods, red meat, salt, sugar, and saturated fat are associated with an elevated risk of cardiovascular disease, type 2 diabetes mellitus, and certain cancer [38–40]. Conversely, healthier dietary patterns, such as the Mediterranean diet, have demonstrated protective effects against NCDs and are associated with increased longevity [41–42]. The global trend towards unhealthy “Western” dietary patterns, characterized by increased intake of processed, high-calorie, and nutrient-deficient foods, has coincided with a rise in obesity, hypertension, and other metabolic disorders [43–44]. This dietary transition has notably contributed to the escalating burden of NCDs, particularly in developing and newly industrialized countries [43].

The frequency and timing of food consumption are related to risk factors for NCDs [45,46]. An increased frequency of eating has been associated with a higher BMI and reduced nutrient density, particularly when a substantial portion of the caloric intake occurs in the evening [47]. In contrast, extended overnight fasting is associated with a relative reduction in BMI [48].

Eating behaviors were evaluated based on the frequency of consumption (1–3 days per month, 1–4 days, or 5–7 days per week) of various food categories, including unhealthy options (non-alcoholic sugary drinks, high-fat foods, and instant foods) and healthy options (fruits and vegetables). Non-alcoholic sugary drinks are characterized by their high sugar content. High-fat foods are recognized by their high fat content. Instant foods are known for their high sodium content. Fruits and vegetables are generally considered healthy foods that are rich in fiber and minerals.

Smoking: Smoking is a major behavioral risk factor for NCDs [49]. Numerous studies have established that individuals who smoke are at an elevated risk of developing cardiovascular disease and lung cancer compared with non-smokers [50]. Smoking behavior was categorized into three groups: non-smokers, former smokers, and current smokers, according to the definitions used in the 2021 Thailand Health Behavior of Population Survey. Non-smokers were identified as individuals who neither smoked at the time of the survey nor had any history of smoking. Former smokers were those who did not currently smoke but had smoked in the past. Current smokers were defined as individuals who reported smoking at the time of the survey. Our smoking variable included not only tobacco consumption but also the use of e-cigarettes and other inhaled agents.

### Statistical analysis

Latent Class Analysis (LCA) was employed to identify unobserved subgroups or classes that exhibited similar multimorbidity patterns [51,52], thereby addressing the heterogeneity of multimorbidity among NCDs within the population [11,12,53]. Specifically, multinomial logistic regression was used to characterize the relationships between the identified multimorbidity patterns and associated risk factors, including age, gender, BMI, living area, dietary behaviors, and smoking status. Age was incorporated as a continuous variable measured in years, whereas the remaining predictors were specified as categorical variables using dummy indicators. This methodological approach is particularly advantageous for identifying distinct risk factors associated with specific multimorbidity patterns and assessing the individual risk of exhibiting a particular multimorbidity pattern.

Evidence from epidemiological studies suggests that the health impacts of key behavioral exposures may differ throughout the life course, reflecting cumulative risk and ageing processes [54–55]. Notably, smoking and sugary beverage consumption have been associated with NCD risk, with potential age-related effect modification [56–57]. To account for plausible life-course heterogeneity in these associations, age-by-exposure interaction terms were specified a priori in the NCD class membership models. Specifically, we included age × smoking behavior (non-smoker, former smoker, and current smoker) and age × non-alcoholic sugary drink frequency (1–3 days/month, 1–4 days/week, and 5–7 days/week). The inclusion of these interactions allows exposure–disease associations to vary with age, rather than assuming constant effects across the population [58]. To maintain model parsimony and limit overparameterization in the multinomial framework, the interaction terms were restricted to these two exposures [56,59].

**Fig 1:**
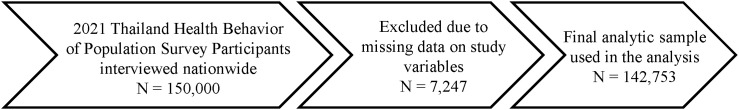
Flow diagram of participant selection.

LCA models were examined and estimated to identify the optimal model using data from the 2021 Thailand Health Behavior of the Population Survey. The study sample comprised 150,000 individuals aged 15–106 years from 84, 000 households across Thailand. After excluding observations with missing data, the final sample comprised 142,753 participants (Fig 1).

The analysis involved specifying, estimating, and selecting the most suitable LCA model using Stata (SE version 18) to determine the appropriate number of latent classes for the multimorbidity of NCDs. Nine prevalent NCDs were considered: hypertension, diabetes, high cholesterol, cardiovascular disease, stroke, chronic respiratory disease, cancer, depression, and osteoarthritis. LCA simultaneously identified the probability of class membership as a function of risk factors such as age, gender, BMI, living area, dietary behaviors, and smoking status.

The LCA was conducted as an exploratory and iterative process, with a progressive increase in the number of classes within the model. The final number of classes was determined based on an evaluation of the model fit indices, classification quality, empirical evidence, and interpretability. Model selection was guided by fit criteria, including the Akaike Information Criterion (AIC) and Bayesian Information Criterion (BIC) [60–61], while entropy was employed to assess classification quality [62], ensuring that the classes were statistically robust and meaningful.

## Results

### Participant characteristics

The analysis included 142,753 participants aged ≥ 15 years, with a mean age of 48.88 years (SD = 17.76). Among these participants, 54.2% were female and 45.8% were male. Approximately half (50.1%) of the participants resided in urban (municipal) areas. The mean BMI was 23.33 (SD = 3.88). In terms of BMI classification, 43.9% were categorized as normal weight, 20.7% as overweight, 27.8% as obese, and 7.7% as underweight. Regarding smoking status, 74.5% were non-smokers, 8.8% were former smokers, and 16.7% were current smokers.

Dietary patterns varied across the sample population. Most participants (50.8%) reported consuming non-alcoholic sugary beverages three or fewer days per month, while 31.1% consumed them 1–4 days per week, and 18.1% consumed them nearly every day (5–7 days per week). The consumption of high-fat foods was prevalent, with 55.1% and 13.9% of participants reporting intakes of 1–4 and 5–7 days per week, respectively. The intake of instant foods was generally low, with 78.7% of the participants consuming them three or fewer days per month, 20.2% consuming them 1–4 days per week, and only 1.0% consuming them almost daily. Fruit and vegetable consumption patterns were more favorable in the present study. Fruit intake was frequent, with 55.5% and 35.6% of participants consuming fruits 1–4 days per week and 5–7 days per week, respectively. Only 8.9% of the participants reported very infrequent fruit consumption (≤ 3 days/month). Similarly, most participants (68.4%) consumed vegetables 5–7 days per week, 28.4% consumed vegetables 1–4 days per week, and 3.2% consumed vegetables ≤ 3 days per month.

On average, participants had 0.49 NCDs (SD = 0.93) per person. The most prevalent conditions were hypertension (19.9%), diabetes mellitus (9.7%), and hyperlipidemia (9.4%). Other conditions included osteoarthritis (4.5%), cardiovascular disease (2.1%), stroke (1.2%), chronic respiratory disease (1.1%), and cancer (0.7%). Depression was reported by 0.4% of participants. Table 1 presents the summary statistics.

**Table 1:** Characteristics of the study population by multimorbidity class.

Characteristics	(N = 142,753)		
	%	Mean	SD
**Age**		48.88	17.76
**Gender**			
Male	45.8%		
Female	54.2%		
**Living area**			
Rural (non-municipal)	49.9%		
Urban (municipal)	50.1%		
**BMI**		23.33	3.88
Underweight	7.7%		
Normal	43.9%		
Overweight	20.7%		
Obese	27.8%		
**Smoking**			
Nonsmoker	74.5%		
Former smoker	8.8%		
Current smoker	16.7%		
**Eating behavior (dietary habit)**			
Non-alcoholic sugary drinks (≤ 3 days/month)	50.8%		
Non-alcoholic sugary drinks (1–4 days/week)	31.1%		
Non-alcoholic sugary drinks (5–7 days/week)	18.1%		
High-fat foods (≤ 3 days/month)	30.9%		
High-fat foods (1–4 days/week)	55.1%		
High-fat foods (5–7 days/week)	13.9%		
Instant foods (≤ 3 days/month)	78.7%		
Instant foods (1–4 days/week)	20.2%		
Instant foods (5–7 days/week)	1.0%		
Fruits (≤ 3 days/month)	8.9%		
Fruits (1–4 days/week)	55.5%		
Fruits (5–7 days/week)	35.6%		
Vegetables (≤ 3 days/month)	3.2%		
Vegetables (1–4 days/week)	28.4%		
Vegetables (5–7 days/week)	68.4%		
**Diseases**			
NCD count		0.49	0.93
Hypertension	19.9%		
Diabetes mellitus	9.7%		
Hyperlipidemia (high cholesterol)	9.4%		
Cardiovascular disease	2.1%		
Stroke	1.2%		
Chronic respiratory diseases	1.1%		
Cancer	0.7%		
Depression	0.4%		
Osteoarthritis	4.5		

### Multimorbidity patterns

In the examination of multimorbidity patterns, latent class models consisting of two to four classes were successfully developed. Models with five or more classes were also investigated; however, they did not converge and were therefore excluded from the analysis. Model fit statistics, including the Akaike information criterion (AIC) and Bayesian information criterion (BIC), progressively decreased as the number of classes increased. The four-class model demonstrated the lowest AIC (350,979) and BIC (352,016) values (Table 2), indicating the best fit among the converged models [61]. The entropy values were 0.81, 0.68, and 0.74 for the two-, three-, and four-class models, respectively, indicating an acceptable classification quality for the selected solution. Although the two-class model exhibited slightly higher entropy, the four-class model provided substantially improved model fit and enhanced clinical interpretability. Considering the statistical fit, entropy, interpretability, and model stability, the four-class solution was identified as the optimal model.

**Table 2:** Goodness-of-fit.

Number of latent classes	Log likelihood	Number of parameters	AIC	BIC	Entropy	Convergence
2	−180334.14	64	360750	361155	0.81	Yes
3	−177470.59	96	355087	355808	0.68	Yes
4	−175384.73	128	350979	352016	0.74	Yes
5	–	–	–	–	–	No

The estimated response probabilities and descriptive characteristics of each class are presented in Table 3. The disease probabilities reflect the estimated response probabilities within each latent class and may differ from the observed disease prevalence derived from the sample. Four distinct latent classes of multimorbidity were identified: low burden, hypertension-metabolic, predominant hypertension, and high multimorbidity, each representing unique patterns of disease clustering and population characteristics.

**Table 3:** Class membership, disease probabilities, and descriptive characteristics of the four latent classes.

	Low burden (N = 88,420)			Hypertension- metabolic (N = 18,222)			Predominant hypertension (N = 35,941)			High multimorbidity (N = 170)		
**Probability**												
Membership probability	58.3%			14.1%			27.5%			0.1%		
				
**Disease probability**												
Hypertension	0.1%			88.9%			26.3%			60.7%		
Diabetes mellitus	0.2%			48.2%			10.0%			65.2%		
Hyperlipidemia (high cholesterol)	0.2%			51.2%			7.1%			78.1%		
Cardiovascular disease	0.1%			7.8%			3.0%			87.6%		
Stroke	0.2%			3.7%			1.7%			93.0%		
Chronic respiratory diseases	0.5%			1.4%			2.1%			89.5%		
Cancer	0.1%			0.8%			1.4%			89.7%		
Depression	0.2%			0.5%			0.5%			93.8%		
Osteoarthritis	0.0%			14.5%			8.3%			95.7%		
**Characteristics**	**%**	**Mean**	**SD**	**%**	**Mean**	**SD**	**%**	**Mean**	**SD**	**%**	**Mean**	**SD**
**Age**		38.90	13.10		66.97	11.22		64.22	10.90		60.66	15.87
**Gender**												
Male	49.1%			34.4%			43.4%			45.9%		
Female	50.9%			65.6%			56.6%			54.1%		
**Living area**												
Rural (non-municipal)	50.1%			47.5%			50.5%			40.0%		
Urban (municipal)	49.9%			52.5%			49.5%			60.0%		
**BMI**		22.97	3.72		24.77	4.27		23.49	3.90		23.71	4.40
Underweight	8.2%			4.4%			8.1%			9.4%		
Normal	48.1%			31.4%			39.8%			40.0%		
Overweight	20.6%			20.1%			21.1%			18.2%		
Obese	23.1%			44.0%			30.9%			32.4%		
**Smoking**												
Nonsmoker	74.9%			77.2%			72.1%			72.4%		
Former smoker	4.2%			15.3%			17.0%			11.2%		
Current smoker	20.9%			7.4%			10.9%			16.5%		
**Eating behavior (dietary habit)**												
Non-alcoholic sugary drinks (≤ 3 days/month)	41.9%			66.3%			64.8%			61.2%		
Non-alcoholic sugary drinks (1–4 days/week)	36.9%			21.4%			21.8%			19.4%		
Non-alcoholic sugary drinks (5–7 days/week)	21.2%			12.3%			13.4%			19.4%		
High-fat foods (≤ 3 days/month)	27.2%			37.0%			37.1%			37.6%		
High-fat foods (1–4 days/week)	57.4%			50.3%			52.0%			52.4%		
High-fat foods (5–7 days/week)	15.4%			12.8%			10.9%			10.0%		
Instant foods (≤ 3 days/month)	72.0%			90.8%			89.1%			87.1%		
Instant foods (1–4 days/week)	26.7%			8.6%			10.4%			12.4%		
Instant foods (5–7 days/week)	1.3%			0.6%			0.6%			0.6%		
Fruits (≤ 3 days/month)	9.1%			7.4%			9.5%			8.8%		
Fruits (1–4 days/week)	57.1%			51.5%			53.5%			53.5%		
Fruits (5–7 days/week)	33.8%			41.1%			37.0%			37.6%		
Vegetables (≤ 3 days/month)	3.3%			2.3%			3.3%			3.5%		
Vegetables (1–4 days/week)	31.8%			21.1%			23.9%			25.3%		
Vegetables (5–7 days/week)	64.8%			76.6%			72.8%			71.2%		
**Disease prevalence**												
NCD count		0.01	0.10		2.43	0.78		0.65	0.60		7.56	1.27
Hypertension	0.0%			94.8%			30.7%			60.0%		
Diabetes mellitus	0.0%			57.6%			9.1%			65.3%		
Hyperlipidemia (high cholesterol)	0.1%			59.3%			6.8%			78.2%		
Cardiovascular disease	0.1%			8.8%			3.3%			88.2%		
Stroke	0.1%			4.2%			1.9%			93.5%		
Chronic respiratory diseases	0.4%			1.4%			2.4%			90.0%		
Cancer	0.1%			0.8%			1.6%			90.0%		
Depression	0.2%			0.5%			0.5%			94.7%		
Osteoarthritis	0.0%			16.1%			9.1%			95.9%		

Class 1 (low burden, 58.3%) was distinguished by an exceptionally low prevalence of all conditions, with probabilities remaining below 1% for all non-communicable diseases (NCDs). Specifically, the probabilities of hypertension (0.1%), diabetes mellitus (0.2%), hyperlipidemia (0.2%), cardiovascular disease (0.1%), stroke (0.2%), chronic respiratory diseases (0.5%), cancer (0.1%), depression (0.2%), and osteoarthritis (0.0%) were notably rare within this class, suggesting a predominantly healthy subgroup. This class comprised the youngest demographic (mean age 38.9 years) and demonstrated a relatively balanced gender distribution (50.9% female). BMI levels were generally lower than those observed in other classes (mean 22.97), with nearly half of the individuals classified as having normal weight (48.1%). Additionally, there was a higher incidence of non-alcoholic sugary drink consumption and instant food intake than in other classes.

Class 2 (hypertension–metabolic, 14.1%) was characterized by a distinct metabolic profile centered on hypertension. The probability of hypertension within this group was remarkably high at 88.9%, with concurrent conditions such as diabetes mellitus (48.2%) and hyperlipidemia (51.2%). Osteoarthritis was present at moderate levels (14.5%), whereas cardiovascular disease (7.8%) and stroke (3.7%) were less prominent. This subgroup comprised the oldest individuals, with a mean age of 66.9 years, and was predominantly female (65.6%). Additionally, this class exhibited the highest BMI levels, with a mean of 24.77, and a considerable proportion of individuals were classified as obese (44.0%).

Class 3 (predominant hypertension, 27.5%) exhibited a hypertension probability of 26.3%, with relatively lower probabilities for other NCDs. The probabilities of diabetes mellitus (10.0%) and hyperlipidemia (7.1%) were notably lower than those observed in Class 2. Other conditions demonstrated even lower probabilities, including cardiovascular disease (3.0%), stroke (1.7%), chronic respiratory diseases (2.1%), cancer (1.4%), depression (0.5%), and osteoarthritis (8.3%), indicating a more limited comorbidity profile. This class comprised older adults with a mean age of 64.2 years and a moderate female predominance (56.6%). The average BMI exceeded the healthy range, with a mean value of 23.49. Additionally, there were notable proportions of individuals who were overweight (21.1%) and obese (30.9%).

Class 4 (high multimorbidity, 0.1%) constituted a small yet clinically critical subgroup characterized by elevated probabilities for nearly all NCDs. The probabilities of disease occurrence were notably high for hypertension (60.7%), diabetes mellitus (65.2%), hyperlipidemia (78.1%), cardiovascular disease (87.6%), stroke (93.0%), chronic respiratory diseases (89.5%), cancer (89.7%), depression (93.8%), and osteoarthritis (95.7%), indicating extensive multimorbidity affecting multiple organ systems. Although this class represented only 0.1% of the study population, it exhibited a distinct multimorbidity profile within the converged four-class solution model. The mean age of individuals in this class was 60.7 years, with a relatively balanced gender distribution (54.1% female). Urban residency was slightly more prevalent in this class (60.0%) than in the other classes.

### Risk factors and multimorbidity

A multinomial latent class regression model was used to investigate the associations between potential risk factors and multimorbidity patterns. The estimation process employed normal weight, male, rural residence, non-smoker, and ≤3 days per month as the reference categories BMI, gender, living area, smoking status, and dietary behaviors, respectively. Table 4 presents the associations between these factors and the multimorbidity classes. The odds of membership in each multimorbidity class were compared with those in the low burden class.

**Table 4:** Risk factors associated with multimorbidity classes: odds ratios and 95% confidence interval.

Variable	Low burden	Hypertension–metabolic	Predominant hypertension	High multimorbidity
**Age**	1	1.21 (1.20, 1.222)***	1.16 (1.15, 1.16)***	1.16 (1.14, 1.19)***
**Gender**				
Female	1	2.02 (1.88, 2.18)***	1.33 (1.23, 1.44)***	1.17 (0.79, 1.73)
**Living area**				
Urban (municipal area)	1	1.14 (1.08, 1.21)***	1.05 (0.99, 1.12)	1.57 (1.15, 2.15)**
**BMI**				
Underweight	1	0.72 (0.62, 0.83)***	1.19 (1.03, 1.37)*	1.61 (0.91, 2.87)
Overweight	1	1.72 (1.60, 1.85)***	1.24 (1.14, 1.34)***	1.06 (0.69, 1.63)
Obese	1	5.27 (4.90, 5.66)***	2.30 (2.13, 2.49)***	2.45 (1.70, 3.55)***
**Smoking**				
Former smoker	1	3.91 (1.98, 7.75)***	2.05 (1.12, 3.77)*	4.52 (0.29, 69.37)
Former smoker x age	1	0.99 (0.98, 1.00)	1.00 (0.99, 1.01)	0.98 (0.94, 1.03)
Current smoker	1	7.90 (4.69, 13.30)***	2.71 (1.79, 4.10)***	29.10 (3.78, 223.83)**
Current smoker x age	1	0.96 (0.95, 0.97)***	0.98 (0.97, 0.98)***	0.94 (0.90, 0.97)***
**Food behavior**				
Non-alcoholic sugary drinks (1–4 days/week)	1	0.49 (0.31, 0.77)**	0.99 (0.69, 1.42)	1.20 (0.16, 9.03)
Non-alcoholic sugary drinks (1–4 days/week) x age	1	1.01 (1.00,1.02)*	1.00 (0.99, 1.00)	0.99 (0.96, 1.02)
Non-alcoholic sugary drinks (5–7 days/week)	1	0.65 (0.39, 1.10)	1.03 (0.67, 1.58)	3.06 (0.38, 24.53)
Non-alcoholic sugary drinks (5–7 days/week) x age	1	1.00 (0.99,1.01)	1.00 (0.99, 1.01)	0.98 (0.95, 1.02)
High-fat foods (1–4 days/week)	1	1.06 (1.00, 1.13)*	1.05 (0.98, 1.12)	0.99 (0.71, 1.39)
High-fat foods (5–7 days/week)	1	1.26 (1.15, 1.38)***	1.04 (0.94, 1.15)	0.86 (0.50, 1.49)
Instant foods (1–4 days/week)	1	1.06 (0.97, 1.15)	1.12 (1.03, 1.22)**	1.07 (0.66, 1.73)
Instant foods (5–7 days/week)	1	2.23 (1.63, 3.05)***	1.64 (1.16, 2.32)**	1.28 (0.18, 9.36)
Fruits (1–4 days/week)	1	1.24 (1.12, 1.38)***	1.06 (0.95, 1.19)	1.16 (0.65, 2.06)
Fruits (5–7 days/week)	1	1.34 (1.20, 1.50)***	1.08 (0.95, 1.21)	1.18 (0.64, 2.16)
Vegetables (1–4 days/week)	1	0.88 (0.72, 1.07)	0.73 (0.59, 0.89)**	0.66 (0.27, 1.60)
Vegetables (5–7 days/week)	1	0.94 (0.78, 1.15)	0.71 (0.59, 0.87)**	0.61 (0.26, 1.44)

*P-value < 0.05, **P-value < 0.01, ***P < 0.001

Age: Age was a significant determinant of class membership. Specifically, each additional year of age was associated with a 21% increase in the likelihood of belonging to the hypertension–metabolic class (OR = 1.21, 95% CI: 1.20–1.22) and a 16% increase in the likelihood of belonging to both the predominant hypertension (OR = 1.16, 95% CI: 1.15–1.16) and high multimorbidity classes (OR = 1.16, 95% CI: 1.14–1.19) compared with the low burden class. These associations were statistically significant across all classes, highlighting age as a robust predictor of class membership.

Gender: Women were significantly more likely than men to be classified within the hypertension–metabolic (OR = 2.02, 95% CI: 1.88–2.18) and predominant hypertension classes (OR = 1.33, 95% CI: 1.23–1.44). No significant differences were observed in the odds of being in the high multimorbidity class compared to the low burden class.

Living area: Place of residence was a significant determinant of class membership. Individuals residing in urban areas were more likely to be categorized within the hypertension–metabolic (OR = 1.14, 95% CI: 1.08–1.21) and high multimorbidity classes (OR = 1.57, 95% CI: 1.15–2.15) than those living in rural areas. No significant differences were identified in the likelihood of being classified into the predominant hypertension class compared to the low burden class.

BMI: Obesity was the strongest BMI predictor of class membership. Individuals classified as obese exhibited substantially higher odds of being categorized within the hypertension–metabolic (OR = 5.27, 95% CI: 4.90–5.66), predominant hypertension (OR = 2.30, 95% CI: 2.13–2.49), and high multimorbidity classes (OR = 2.45, 95% CI: 1.70–3.55). Overweight participants demonstrated significantly increased odds of membership in the hypertension–metabolic (OR = 1.72, 95% CI: 1.60–1.85) and predominant hypertension classes (OR = 1.24, 95% CI: 1.14–1.34), but not in the high multimorbidity class. Underweight status was associated with reduced odds of belonging to the hypertension–metabolic class (OR = 0.72, 95% CI: 0.62–0.83) but increased odds of belonging to the predominant hypertension class (OR = 1.19, 95% CI: 1.03–1.37). No statistically significant association was found in the high multimorbidity class.

Smoking status: Smoking is a significant risk factor for multimorbidity. Former smokers exhibited substantially increased odds of being classified within the hypertension–metabolic class (OR = 3.91, 95% CI: 1.98–7.75) and predominant hypertension class (OR = 2.05, 95% CI: 1.12–3.77). No significant association was observed in the high multimorbidity class. Current smokers showed the most pronounced associations. Compared with non-smokers, current smokers had nearly eight times higher odds of membership in the hypertension–metabolic class (OR = 7.90, 95% CI: 4.69–13.30), almost three times higher odds of predominant hypertension (OR = 2.71, 95% CI: 1.79–4.10), and markedly elevated odds of belonging to the high multimorbidity class (OR = 29.10, 95% CI: 3.78–223.83). The interaction terms indicated that the effect of current smoking slightly diminished with increasing age across all multimorbidity classes, whereas no significant age interaction was observed among former smokers.

Non-alcoholic sugary drinks: Moderate consumption of non-alcoholic sugary drinks (1–4 days per week) was associated with a reduced likelihood of classification within the hypertension–metabolic class (OR = 0.49, 95% CI: 0.31–0.77). No significant associations were observed for the predominant hypertension or high multimorbidity classes. The interaction between moderate consumption of sugary beverages and age was statistically significant (OR = 1.01, 95% CI: 1.00–1.02), suggesting that this association exhibited a slight variation with increasing age. Frequent consumption (5–7 days per week) was not significantly associated with class membership.

High-fat food consumption: The consumption of high-fat foods was positively associated with the hypertension–metabolic class. Moderate intake (1–4 days/week) was associated with a slight increase in the probability of class membership (OR = 1.06, 95% CI: 1.00–1.13), whereas frequent intake (5–7 days/week) was associated with a 26% increase in the odds (OR = 1.26, 95% CI: 1.15–1.38). No significant associations were identified for the predominant hypertension or high multimorbidity classes.

Instant food consumption: A similar positive relationship was observed for instant food consumption. Participants who moderately consumed instant foods (1–4 days per week) had an increased likelihood of being classified within the predominant hypertension class (OR = 1.12, 95% CI: 1.03–1.22). Moreover, frequent consumption (5–7 days per week) significantly increased the probability of classification in both the hypertension–metabolic (OR = 2.23, 95% CI: 1.63–3.05) and predominant hypertension classes (OR = 1.64, 95% CI: 1.16–2.32). No significant association was found in the high multimorbidity class.

Fruit consumption: Fruit consumption was positively correlated with the hypertension–metabolic class. Specifically, individuals who consumed fruits 1–4 days per week had a 24% higher likelihood of being classified in this class (OR = 1.24, 95% CI: 1.12–1.38), whereas those who consumed fruits 5–7 days per week exhibited a 34% increased likelihood (OR = 1.34, 95% CI: 1.20–1.50). Importantly, fruit consumption was not significantly associated with the predominant hypertension or high multimorbidity classes.

Vegetable consumption: Vegetable consumption decreased the probability of membership in the predominant hypertension class. Moderate vegetable consumption (1–4 days/week) was associated with a 27% reduction in the likelihood of belonging to this class (OR = 0.73, 95% CI: 0.59–0.89), whereas frequent consumption (5–7 days/week) was associated with a 29% reduction (OR = 0.71, 95% CI: 0.59–0.87). No statistically significant associations were found for the hypertension–metabolic or high multimorbidity classes.

## Discussion

The largest class, comprising 58.3% of the population, was classified as the low burden class and represented a relatively healthy demographic with a minimal prevalence of all examined conditions. The remaining 41.7% of the population exhibited varying degrees of multimorbidity, posing eminent health and economic challenges for Thailand and reflecting disparities across age, gender, living location, BMI, smoking status, and dietary behaviors. The hypertension–metabolic class highlighted the growing burden of metabolic-related conditions, with particularly high probabilities of hypertension (88.9%), hyperlipidemia (51.2%), and diabetes mellitus (48.2%). In contrast, the predominant hypertension class demonstrated a more limited comorbidity profile, characterized mainly by hypertension (26.3%) and comparatively lower probabilities of diabetes (10.0%) and hyperlipidemia (7.1%), although chronic respiratory diseases (2.1%) and cancer (1.4%) were slightly more prevalent than those observed in the hypertension-metabolic class. The high multimorbidity class illustrated the convergence of nearly all NCDs within a small subgroup (0.1%), with very high probabilities across conditions, underscoring the need for integrated and multidisciplinary care approaches for individuals with complex multimorbidity.

### Risk factors and their implications

This study determined that age was significantly associated with multimorbidity, corroborating the findings of Feng et al. [63]. Consistent with the study by Park, Lee, and Park [64], women were more frequently represented in the hypertension–metabolic class. Our research further indicated that in 2021, Thai women were more commonly classified within multimorbidity subgroups, a finding that contrasts with the results of Feng et al. [63], whose study demonstrated that in 2005, Thai men exhibited a higher prevalence of such conditions than women. Collectively, these findings suggest a shift in gender distribution patterns over the past 16 years, highlighting the evolving disparities in health profiles and associated economic burdens in Thailand. Furthermore, urban residents were more commonly observed in the hypertension–metabolic class, consistent with prior studies [17], suggesting that urban environments are characterized by distinct patterns of health. Spatial analyses from the 2005 Thai Cohort Study [53] similarly reported a higher prevalence of multimorbidity in more developed areas.

Consistent with previous studies [34,65,66], the maintenance of a healthy BMI was associated with membership in the low burden class, whereas overweight and obesity were more frequently observed in the hypertension–metabolic and high multimorbidity classes. Notably, obesity was strongly associated with the hypertension–metabolic class, which was characterized by a high prevalence of hypertension, diabetes mellitus, and hyperlipidemia. This pattern corroborates evidence supporting a close relationship between elevated BMI and cardiometabolic risk [67].

Furthermore, underweight status was distinctly distributed across the identified disease patterns. Individuals with underweight status were less likely to belong to the hypertension–metabolic class and more frequently classified in the predominant hypertension class. This finding aligns with studies indicating that lean or underweight individuals more often correspond with isolated hypertension or metabolically healthier profiles rather than with metabolic-syndrome–dominant clusters [68–70]. Evidence from low-BMI diabetes populations and cardiovascular registries further suggests that lean individuals may exhibit hypertension with fewer concurrent metabolic abnormalities, reflecting a pattern that is distinct from obesity-related metabolic clustering [71–72]. Collectively, these findings support the heterogeneous distribution of BMI across multimorbidity subgroups in the Thai population.

Smoking behavior, including the use of tobacco products, e-cigarettes, and inhaled substances, is the most detrimental risk factor for multimorbidity. Current smokers showed significantly increased odds across multimorbidity classes, with a substantially greater risk than former smokers. This risk pattern is concerning given the rising popularity of e-cigarettes among Thai youth despite their illegal status, with usage rates increasing from 3.3% in 2015 to 17.6% in 2022 [73]. The overall adult smoking prevalence remains high at approximately 19% and has declined modestly over the past two decades [74]. Thailand faces escalating health and economic challenges due to smoking-related harm to its population. Tobacco use imposes substantial healthcare costs and reduces the productivity of the user. The increasing prevalence of e-cigarette use, particularly among younger populations, threatens to exacerbate the burden on the healthcare system. Enforcement measures, prevention programs, and public education initiatives are urgently required.

In this study, the dietary factors demonstrated distinct patterns across the identified classes. Dietary behaviors were measured based on self-reported frequency of consumption rather than portion size or total intake. A higher intake of high-fat and instant foods was more frequently observed among individuals in the hypertension–metabolic and predominant hypertension classes, consistent with evidence linking ultra-processed and high-fat diets to cardiometabolic abnormalities and elevated blood pressure [75–76]. Conversely, vegetable consumption was more prevalent in the low burden class and less represented in the predominant hypertension class, aligning with research indicating that increased vegetable intake is associated with more favorable blood pressure profiles [77–78].

The relationship between sugary beverage consumption and multimorbidity varied by class and age. Moderate consumption (1–4 days/week) was less prevalent in the hypertension–metabolic class than in the low burden class and was associated with a reduced likelihood of membership in the hypertension-metabolic class compared to the low burden class. However, the interaction with age demonstrated that this association diminished with increasing age. This pattern reflects age-related dietary habits, as older individuals, who were more likely to belong to the hypertension–metabolic class, reported a lower intake of sugary beverages. These findings align with evidence indicating that sugary drink consumption decreases with age, potentially due to changes in taste preferences, dietary modifications following diagnosis, and heightened health awareness [79–80].

However, our findings contrast with those of previous studies that have identified protective associations between fruit consumption and multimorbidity, particularly within broader plant-based dietary patterns [81]. In this study, a higher intake of fruit was more frequently observed in the hypertension–metabolic class than in the low burden class. While fruit consumption is generally considered beneficial, emerging evidence suggests that a high intake, especially of fruits with higher natural sugar content, may be associated with less favorable cardiometabolic profiles in certain populations [82–83].

The findings underscore the intricate nature of multimorbidity patterns in Thailand, which are characterized by variations in age, gender, BMI, dietary and smoking behaviors, and urban-rural residence. This is consistent with previous studies demonstrating associations between multimorbidity patterns and demographic, behavioral, and socioeconomic characteristics [3,26,64]. The identification of distinct multimorbidity patterns offers a more comprehensive understanding than mere disease counts, highlighting the heterogeneity of health profiles within a population. This analysis contributes additional evidence of disease clustering in Southeast Asia, where empirical data remain limited. These findings highlight important public health considerations related to population aging, urban–rural disparities, healthy weight management, tobacco control, and nutritional education.

Despite these insights, this study has some limitations. First, the cross-sectional design precluded the temporal assessment of transitions between multimorbidity subgroups. Consequently, the findings represent multimorbidity patterns at a single point in time, which may change as population demographics, behavioral and lifestyle factors, and environmental conditions evolve over time. Second, owing to the complexity inherent in mixture modeling, survey design factors, including stratification, clustering, and sampling weights, were not incorporated into the latent class analysis (LCA). Consequently, the reported class percentages describe the distribution within the analyzed dataset rather than the exact national population estimate. Third, reliance on self-reported data may introduce recall or reporting biases.

Future longitudinal studies are necessary to examine the transitions between multimorbidity subgroups and enhance the understanding of the biological, behavioral, and healthcare-related processes underlying these patterns. Further research should explore additional lifestyle factors, such as physical activity and alcohol consumption, as well as potential synergistic interactions between behavioral risk factors (e.g., smoking and high sugar dietary patterns). Investigating the interactions between infectious and non-communicable diseases, including HIV-associated multimorbidity, may provide further insights into the evolving burden of multimorbidity in Thailand. Comparative studies across Southeast Asian and other countries would also help elucidate whether similar multimorbidity patterns emerge across different populations and health system contexts.

In the context of population aging and evolving smoking patterns, including the increasing use of e-cigarettes among the youth, the prevalence and composition of multimorbidity subgroups in Thailand may continue to evolve. Addressing these challenges through integrated and gender-sensitive public health strategies may aid in managing the growing complexity of multimorbidity in Thailand.

## Conclusion

This study offers one of the most comprehensive analyses of multimorbidity patterns in Thailand, employing a large, nationally representative sample and latent class analysis. By investigating the characteristics and modifiable behavioral factors, this study provides further evidence of the clustering of non-communicable diseases within the Thai population.

Our findings show that age, gender, BMI, smoking, dietary behaviors, and urban-rural residence were differentially associated with the four identified multimorbidity subgroups, highlighting the heterogeneity of disease clustering in the population. This study contributes to the global evidence base by illustrating how multimorbidity patterns differ across demographic characteristics, lifestyle behaviors, and urban–rural residences.

In summary, the identification of distinct multimorbidity subgroups provides a structured framework for understanding disease clustering in an aging population. These findings may inform public health planning by supporting population-specific and context-sensitive approaches to multimorbidity in Thailand and similar settings.
